# Continuous and noninvasive hemoglobin monitoring reduces red blood cell transfusion during neurosurgery: a prospective cohort study

**DOI:** 10.1007/s10877-015-9660-4

**Published:** 2015-02-04

**Authors:** Wael N. Awada, Maher F. Mohmoued, Tarek M. Radwan, Gomaa Z. Hussien, Hany W. Elkady

**Affiliations:** Department of Anesthesia, ICU and Pain Management, Cairo University, Manyal, Cairo, Egypt

**Keywords:** Hemoglobin, Noninvasive monitoring, Transfusion

## Abstract

Continuous, noninvasive hemoglobin (SpHb) monitoring provides clinicians with the trending of changes in hemoglobin, which has the potential to alter red blood cell transfusion decision making. The objective of this study was to evaluate the impact of SpHb monitoring on blood transfusions in high blood loss surgery. In this prospective cohort study, eligible patients scheduled for neurosurgery were enrolled into either a Control Group or an intervention group (SpHb Group). The Control Group received intraoperative hemoglobin monitoring by intermittent blood sampling when there was an estimated 15 % blood loss. If the laboratory value indicated a hemoglobin level of ≤10 g/dL, a red blood cell transfusion was started and continued until the estimated blood loss was replaced and a laboratory hemoglobin value was >l0 g/dL. In the SpHb Group patients were monitored with a Radical-7 Pulse CO-Oximeter for continuous noninvasive hemoglobin values. Transfusion was started when the SpHb value fell to ≤l0 g/dL and was continued until the SpHb was ≥l0 g/dL. Blood samples were taken pre and post transfusion. Percent of patients transfused, average amount of blood transfused in those who received transfusions and the delay time from the hemoglobin reading of <10 g/dL to the start of transfusion (transfusion delay) were compared between groups. The trending ability of SpHb, and the bias and precision of SpHb compared to the laboratory hemoglobin were calculated. Compared to the Control Group, the SpHb Group had fewer units of blood transfused (1.0 vs 1.9 units for all patients; *p* ≤ 0.001, and 2.3 vs 3.9 units in patients receiving transfusions; *p* ≤ 0.0 l), fewer patients receiving >3 units (32 vs 73 %; *p* ≤ 0.01) and a shorter time to transfusion after the need was established (9.2 ± 1.7 vs 50.2 ± 7.9 min; *p* ≤ 0.00 l). The absolute accuracy of SpHb was 0.0 ± 0.8 g/dL and trend accuracy yielded a coefficient of determination of 0.93. Adding SpHb monitoring to standard of care blood management resulted in decreased blood utilization in high blood loss neurosurgery, while facilitating earlier transfusions.

## Introduction

Red blood cell (RBC) transfusions are initiated to maintain oxygen transport and sustain life but transfusion practices vary widely by hospital, procedure, and physician [1, 2]. Observational studies associate RBC transfusion with risk in the form of postoperative infection [3], impaired pulmonary function [4], and increased length of stay and mortality [5, 6]. Meta-analyses of randomized controlled trials indicate that restrictive transfusion practices are safe and may provide benefit [7]. Additionally, RBC transfusion is costly and a significant contributor to the expense of surgical care [8].

These facts are balanced by the knowledge that anemia, common amongst surgical patients, is also independently associated with adverse outcomes [9]. Therefore, balancing the need for RBC transfusion to mitigate anemia with the risks associated with administration of blood products can be a complicated aspect of care during and after surgery. Laboratory hemoglobin (Hb) values are a primary indicator for the need for blood transfusion [10], but are only available intermittently and results can be delayed in the period between blood draw and laboratory analysis. This means that during surgery, initial and subsequent transfusion decisions are often made without recent Hb results [1]. In addition to laboratory Hb values, point-of-care devices such as the HemoCue (HemoCue, Angelhom, Sweden) can be used in the operating room to detect Hb values; however, this point-of-care device is invasive, and requires intermittent blood sample analysis. The HemoCue also lacks the ability to provide real-time trends, hence limiting its use in guiding blood management decisions [11]. Continuous monitoring of vital signs and estimated blood loss help guide transfusion decisions in the absence of Hb; however, these measures can be inaccurate and misleading [12–14], leading to calls for real-time guidance of blood loss [13].

Continuous and noninvasive hemoglobin (SpHb) monitoring is now possible with Pulse CO-Oximetry technology and multi-wavelength sensors, which also provide traditional pulse oximetry measurements. SpHb monitoring provides real-time trends in the values of hemoglobin, indicating stable hemoglobin when it may be perceived to be dropping and rising hemoglobin when it may be perceived as not rising fast enough. SpHb monitoring has been shown to help anesthesiologists reduce RBC transfusion frequency and average units transfused per patient in a randomized controlled trial in moderate to low blood loss orthopedic surgery [15]. The objective of this study was to evaluate SpHb monitoring impact on RBC transfusions in high blood loss surgery. We hypothesized that the additional information provided by SpHb monitoring would reduce red blood cell transfusion by preventing over transfusion and decrease the time to start transfusions.

## Materials and methods

### Eligibility criteria

The protocol was approved by the Medical Research Ethics Committee of Cairo University Faculty of Medicine. The ethics committee waived the requirement for written informed consent and considered the general surgical informed consent to be sufficient for the study, given Egyptian cultural considerations and the noninvasive nature of monitor and sensors. Patient charts were screened the day prior to scheduled neurosurgery for inclusion and exclusion criteria. A convenience sample of patients who met the criteria was included in a prospective trial conducted between February and August, 2012 in a tertiary care, academic medical center. Adult patients between 15 and 60 years and scheduled to undergo neurosurgical procedure were eligible for the study if they had an American Society of Anesthesiologists (ASA) physical status of I–II. Exclusion criteria included significant liver disease defined as serum alanine aminotransferase and serum aspartate aminotransferase >2.5 times normal, significant renal disease defined as serum creatinine >1.5 mg/dl or creatinine clearance <40 ml/min, pregnancy, significant coagulopathy defined as international normalized ratio >1.5, use of antiplatelets or anticoagulants, anemic patients with Hb concentrations less than 10 g/dL, patients scheduled for procedures with expected low blood loss such as level 1 laminectomy, shunts, nerve decompression, microdiscectomy, etc., and, any patient in whom simultaneous SpHb and Hb measurements could not be obtained.

### Study design

In this prospective cohort study design, given the limitations on the availability of the device and the number of sensors that were available to use, only one patient scheduled for neurosurgery the next day was randomly selected using the sealed envelope method to be in the SpHb Group. The remaining patients on that same day were enrolled into a standard care group (Control Group). Envelopes were prepared each day to match the number of enrolled patients. Prior to surgery, patient demographic information (gender, age, estimated weight, ASA status) was recorded. All patients received standard-of-care perioperative monitoring and anesthesia per ASA guidelines by the primary anesthesia care provider.

### Study procedures

**Fig. 1:**
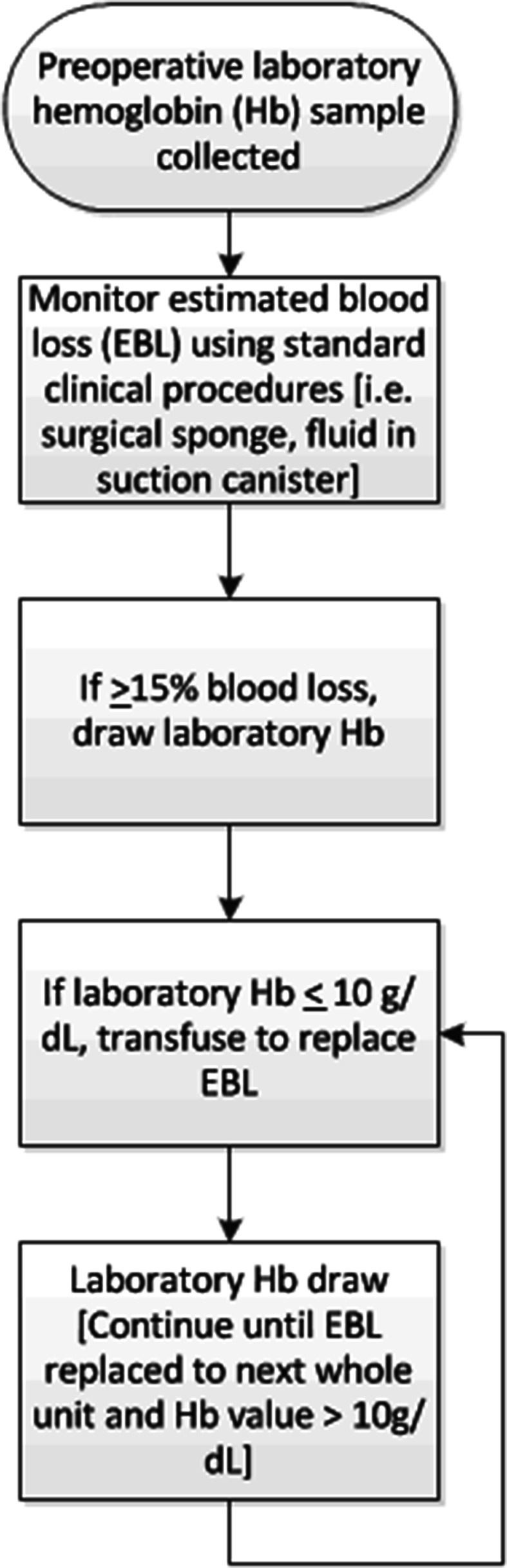
Flow diagram of Hb laboratory sample draws

Patients in the Control Group received intraoperative Hb monitoring by intermittent blood sampling analyzed by the central laboratory. An initial blood sample was taken prior to surgery (baseline Hb) in all patients. Intra-operatively, blood samples were taken when estimated blood loss (EBL) was ≥15 % of total blood volume (Fig. 1). In the SpHb Group, besides ASA monitoring and laboratory Hb, patients were monitored with a Radical-7 Pulse CO-Oximeter, v7748, connected to a R2-25 adult ReSposable sensor (Revision E, Masimo, Irvine, CA) that displayed continuous SpHb values. After induction of anesthesia, the SpHb sensor was placed on the ring finger of the non-dominant hand contralateral to the arterial line with the emitter and detector of the sensor properly aligned per the manufacturer’s directions for use. In both groups transfusion was started when Hb was ≤l0 g/dL, was continued until the EBL was replaced to the next whole unit, and an Hb value was confirmed to be >l0 g/dL. A blood sample for laboratory analysis was taken prior to surgery (baseline Hb), before each transfusion was started, and again when it was discontinued. The Hb and SpHb values were recorded on a case report form at baseline and before and after any packed RBC transfusion.

Blood sampling technique was the same for both groups. Arterial blood was drawn from a 20 gauge radial artery cannula into 2 mL ethylenediaminetetraacetic acid collection tubes, thoroughly mixed then sent immediately to the central lab for analysis by a single hematology analyzer (GEN-S hematology analyzer, Beckman-Coulter Inc., Brea CA). The hematology analyzer was calibrated daily according to the manufactures directions for use and good laboratory practice. All samples were processed and laboratory values returned according to the standard practice of the hospital. The variables recorded for each patient included the time of each blood draw, the start time of each transfusion, and the number of units of blood transfused during the intraoperative period.

All anesthesiologists who participated in the study treated patients in both groups. Blood loss for each patient was estimated by the number of saturated sponges or surgical gauze, the amount of suctioned blood in the waste canister, and the amount of blood in the surgical field. Total blood volume was estimated by multiplying the patient’s weight by 75 mL/kg.

### Data analysis

#### Sample size calculation

Historically, at our institution, it has been observed that patients necessitating a blood transfusion would require an average of 1.8 units of blood. Therefore, in order to detect a 33 % reduction in red blood cell units transfused with the addition of SpHb monitoring (expected effect size equal to 0.6 units) with a standard deviation of 1.0 unit, and assuming a α risk of 0.05 and a β risk of 0.20, 45 subjects would be required for each group for a total of 90 patients. Assuming 10 % of enrolled patients would not complete the study we targeted enrollment of a minimum of 99 patients.

#### Effect on transfusion

The following transfusion variables were calculated and compared between the SpHb Group and Control Group: (a) average baseline Hb; (b) percent of patients transfused; (c) average amount of blood transfused; (d) average change in Hb from pre- to post-transfusion, and average amount over or under hemoglobin target post-transfusion; (e) average amount of blood transfused in those who received transfusions; (f) number of patients that received 3 or more units of RBCs; (g) the average time between taking the hemoglobin sample or SpHb measurement, and the start of transfusion (transfusion delay); and, (h) the average total blood loss.

#### SpHb absolute and trend accuracy

To assess absolute accuracy, or single point comparison, paired SpHb and Hb measurements were compared pre- and post-transfusion and bias and standard deviation were calculated. A repeated measures Bland–Altman graph with limits of agreement (1.96 × standard deviation, adjusted for the bias) was plotted to show agreement across the range of values. To assess trending accuracy, a regression plot of changes in Hb and corresponding changes in SpHb was plotted and a coefficient of determination (R^2^) was calculated.

For evaluation of differences between groups, two proportion Z-tests, two sided t-tests or Mann–Whitney rank sum tests for non-normally distributed data were performed, as appropriate, with a *p* value of <0.05 considered statistically significant.

## Results

### Demographics

A total of 111 patients were enrolled with five patients excluded due to protocol deviations: three in the Control Group due to massive blood loss requiring transfusion to start before the laboratory result was returned and two in the SpHb Group due to the time delay between taking the baseline Hb and the baseline SpHb.

**Fig. 2:**
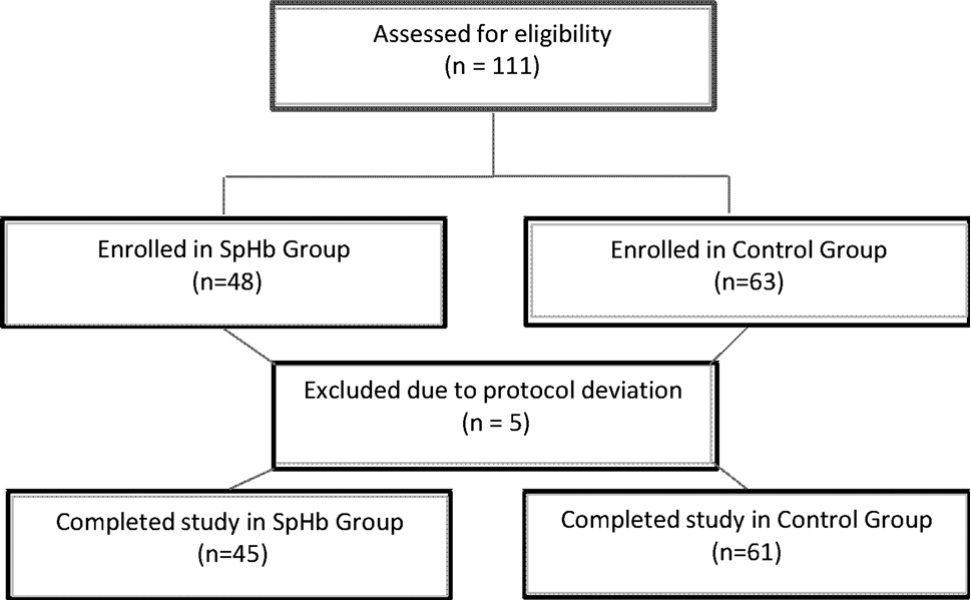
Flow diagram of screened and included patients

The 106 patients who completed the study were 53 % female and had an average age of 37.6 ± 14.1 (mean ± SD). A total of 45 patients were included in the SpHb Group and 61 patients were included in the Control Group. A flow diagram of the patient allocation is shown in Fig. 2. Patients were scheduled for various neurosurgical procedures such as frontal, temporal or occipital glioma excisions, meningioma excisions and frontal, temporal and occipital mass excisions. The proportion of surgery types was similar for both groups. Patient demographics are shown in Table 1 with no significant differences between groups except the SpHb group was lower in weight.

**Table 1:** Patient characteristics

	Control group	SpHb group	p value
No. of patients	61	45	–
Gender (M/F) (%)	49/51	44/55	–
Age range (y)	13–60	12–60	0.66
Weight, range (kg)	44–76	42–74	0.03
ASA Status I/II (%)	78/21	64/35	0.11
Procedures n (%)	61	45	
Glioma excision	12 (20)	11 (24)	
Meningioma excision	11 (18)	10 (22)	
Frontal/temporal/occipital mass excision	4 (7)	4 (9)	
Other (e.g. frontal lobectomy, depressed compound skull fracture, cerebral vascular anastomosis, pituitary adenoma)	34 (56)	20 (44)	

Demographics of 106 neurosurgery patients allocated to Control Group (standard care hemoglobin monitoring) or SpHb Group (standard of care plus SpHb monitoring) for blood management

*SD* standard deviation

### Impact on transfusion

Transfusion variables are shown in Table 2. The SpHb Group had mean baseline hemoglobin of approximately 1 g/dL lower than the Control Group. The percentage of patients transfused in each group was not different, but there were fewer RBC units transfused in the SpHb Group versus the Control Group over all patients (1.0 vs 1.9 units; *p* ≤ 0.00 l) and in patients receiving transfusions (2.3 vs 3.9 units; *p* ≤ 0.0 l). The SpHb Group also had a lower percentage of transfused patients receiving >3 RBC units (32 vs 73 %; *p* ≤ 0.01). After RBC transfusion, the SpHb had a lower Hb increase after RBC transfusion was initiated (1.8 ± 0.9 vs 2.6 ± 1.2 g/dL; *p* ≤ 0.05) and shorter time to transfusion after transfusion need was established (9.2 ± 1.7 vs 50.2 ± 7.9 min; *p* ≤ 0.00 l).

**Table 2:** Transfusion variables

	Control group (n = 61)	SpHb group (n = 45)	p value
Baseline Hb (mean ± SD, g/dL)	12.4 ± 1.6	11.5 ± 1.0	0.02
Total blood loss (mean ± SD, mL)	1,807 ± 794	1,732 ± 804	0.30
Percent blood loss (total blood loss/estimated total blood volume) (%)	21.7 ± 16.4	27.7 ± 25.8	0.18
Pre-transfusion Hb (mean ± SD, g/dL)	8.3 ± 1.2	8.6 ± 1.3	0.23
Post-transfusion Hb (mean ± SD, g/dL)	10.8 ± 0.5	10.3 ± 0.5	<0.01
Change in Hb, pre-transfusion to post-transfusion (mean ± SD, mL)	2.6 ± 1.2	1.8 ± 0.9	<0.05
Patients transfused, n (%)	30 (49)	19 (42)	0.61
RBC units transfused per patient over all patients (mean ± SD)	1.9 ± 2.3	1.0 ± 1.5	0.01
RBC units transfused per transfused patient (mean ± SD, units)	3.9 ± 1.7	2.3 ± 1.5	<0.01
Transfused patients receiving >3 RBC units (%)	73	32	<0.01
Transfusion delay from determination of need (min)	50.2 ± 7.9	9.2 ± 1.7	<0.001

Transfusion variables for neurosurgery patients in Control Group (standard care hemoglobin monitoring and SpHb Group (SpHb monitoring) for blood management

*SD* standard deviation

### Assessment of accuracy

**Fig. 3:**
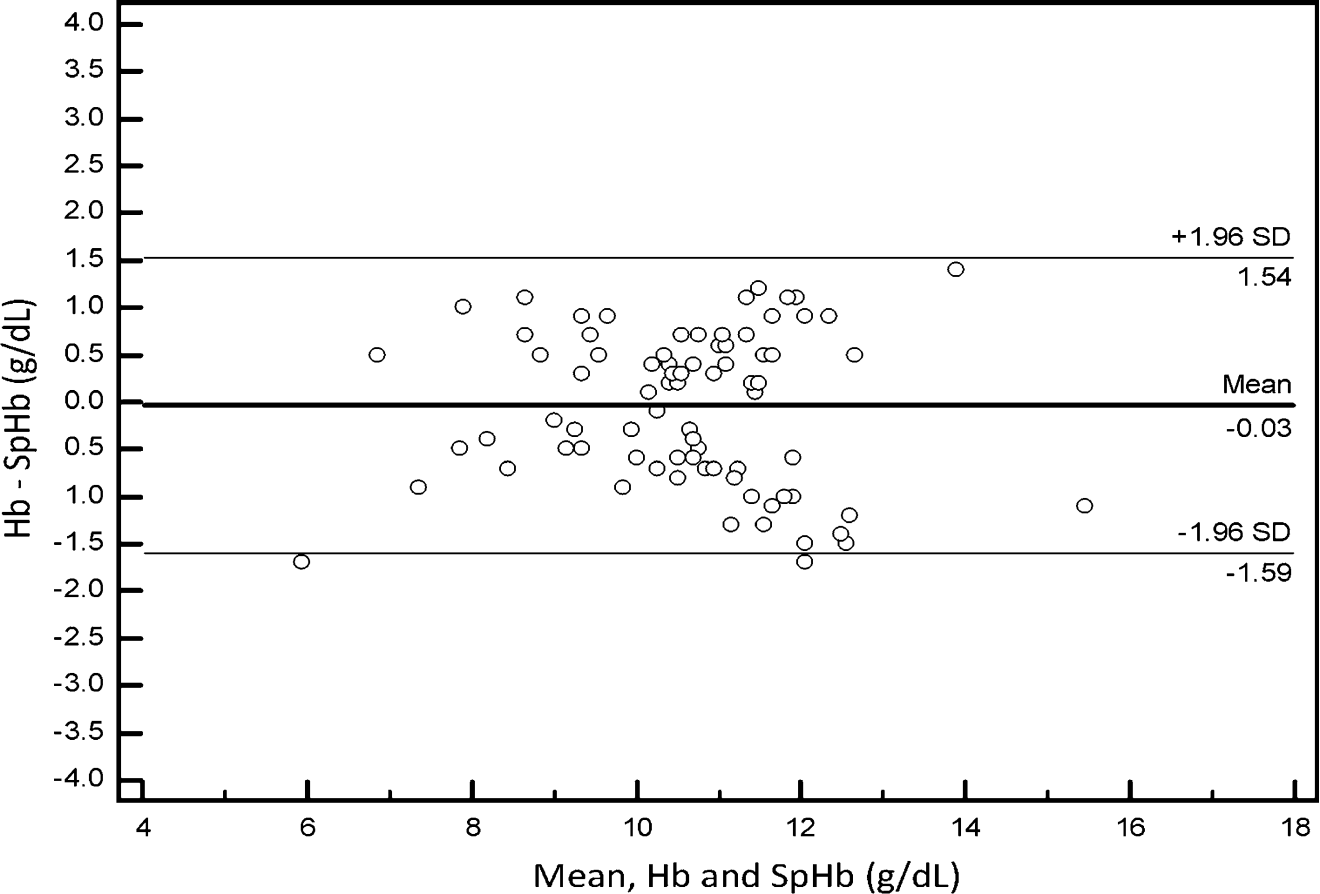
Bland and Altman plot of 83 SpHb and Hb data pairs collected from 45 neurosurgery patients, showing bias (*solid line*) and limits of agreement (*dashed line*)

For assessment of absolute accuracy, 83 SpHb and Hb comparisons were made (45 baseline, 19 pre-transfusion, and 19 post-transfusion). The bias and standard deviation was 0.0 ± 0.8 g/dL over all comparisons, 0.1 ± 0.9 g/dL for baseline Hb, −0.1 ± 0.8 g/dL for pre-transfusion Hb and −0.1 ± 0.5 g/dL for post-transfusion Hb values. The Bland–Altman plot for all values showed limits of agreement of −1.6 to 1.5 g/dL (Fig. 3).

**Fig. 4:**
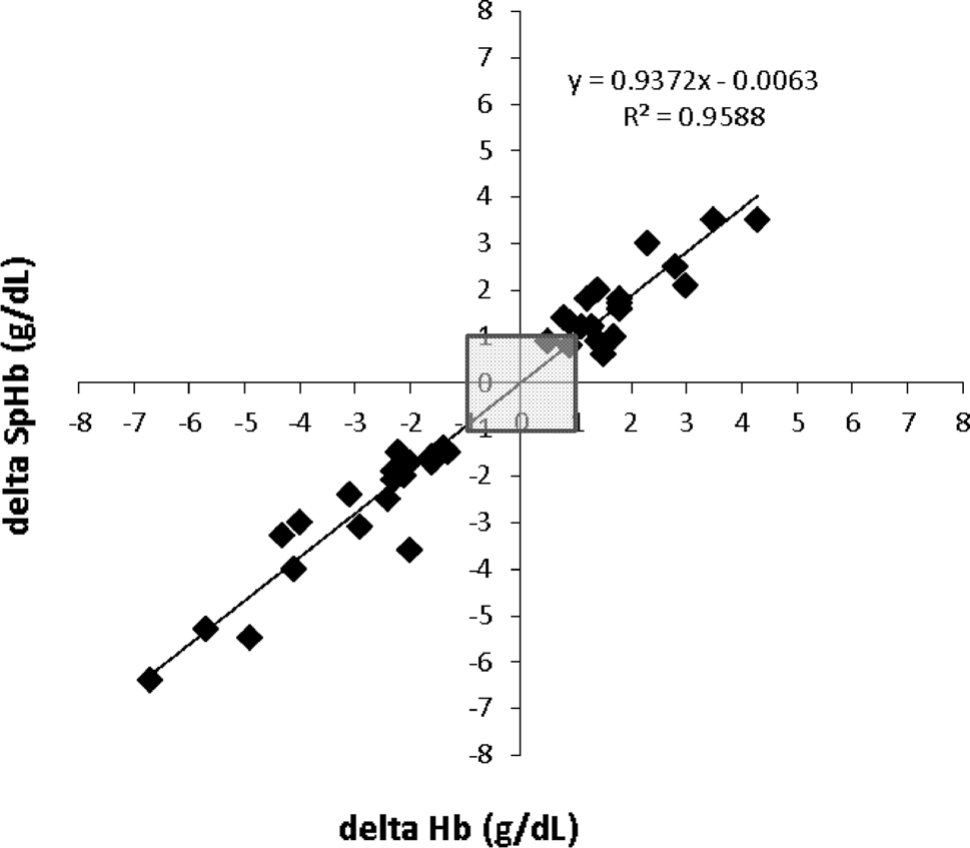
Regression plot of directional changes in consecutive SpHb values (delta SpHb, Y axis) compared to consecutive changes in Hb values (delta Hb, X axis), collected from 45 neurosurgery patients. Data points within the *shaded area* are below the clinically relevant threshold of changes of ≤1 g/dL

Trend accuracy analysis (change in consecutive SpHb values compared to changes in time-matched consecutive Hb values) showed a coefficient of determination (R^2^) of 0.96 (Fig. 4).

## Discussion

Over 90 million times per year around the world, a physician makes the decision to transfuse blood [16]. Since blood is an organ itself, this “liquid organ transplant” is provided with good intentions and is billed as the “gift of life”. However, growing data indicate that blood may not always be helpful to the patient and in fact, may expose the patient to unwarranted risks, and hospitals and/or health care systems to unnecessary costs.

Post-operatively, continuous hemoglobin trending could provide added benefit by indicating hemorrhage that is otherwise not apparent. This trending approach aligns well with The Joint Commission’s call on hospitals to track appropriateness of transfusions as a quality indicator [17]. Further, the American Medical Association and The Joint Commission have targeted RBC transfusions as one of the top five procedures in medicine for “overuse”, defined as using a treatment where the likelihood of benefit is negligible and the patient is exposed to risk of harm [18].

In this study, our primary aim was to determine how a new tool, SpHb monitoring, may impact transfusion decision making in high blood loss surgery when added to standard practice. Our study showed that patients at risk for high blood loss who received SpHb monitoring received fewer RBC transfusions, on average. In this study, there were no differences in percentage of patients transfused or mean blood loss between the Control and SpHb Groups, indicating that patients and procedures in both groups were similar.

The baseline hemoglobin of the SpHb Group was approximately 1 g/dL lower than the Control Group. Multiple studies have shown that a lower baseline hemoglobin significantly increases the likelihood of receiving a RBC transfusion [19]. Therefore, the SpHb group should have been more likely to receive a RBC transfusion in the study. In our study, we observed no difference in the overall transfusion rate (49 % in the Control Group, 42 % in the SpHb Group) but we did observe a large difference in the number of multi-unit transfusions, leading to a significantly lower average units transfused in the SpHb group. If both groups in our study had the same baseline hemoglobin, it is possible that we might have also observed a difference in the overall transfusion rate, as was previously shown in a randomized trial of SpHb monitoring in lower blood loss orthopedic surgery, in which the RBC transfusion rate was 4.5 % in the Control Group and 0.6 % in the SpHb Group [15].

In contrast, the lower average RBC units transfused in our study were primarily due to the differences in multi-unit transfusions, as 32 % of the patients in the SpHb Group received three or more RBC units versus 73 % in the Control Group. This may have occurred due to the ability of the anesthesiologist to gauge in real-time that the transfusion had achieved the desired, increased effect on hemoglobin levels, therefore preventing the decision to have additional RBC units transfused. Further, the real time assessment also affected the initial decision to transfuse, resulting in a quicker decision to initiate a transfusion when needed due to a lack of delay in laboratory Hb values. It is unlikely that the full benefit of continuous monitoring could be achieved by a more diligent assessment of Hb levels using standard procedures. When using laboratory values, the inherent lag time from the request of a test to obtaining the result would be too great, and using an invasive point-of-care device would still require clinician initiative to request a test, therefore, unexpected changes, or unexpected stabilization of hemoglobin, would not be detected.

It is important to note that although the post-transfusion Hb was different between groups with the Control Group being higher, the mean post-transfusion hemoglobin of the SpHb Group was not in the anemic range of <10 g/dL. Anemia is a risk factor for surgical patients, but may be of a special concern to neurosurgical patients. Subarachnoid Haemorrhage [20], traumatic brain injury [21], and other neurosurgical patients [22] have worse outcomes if they are anemic.

As a secondary aim, we studied the absolute accuracy and trend accuracy of SpHb. The accuracy of SpHb has been evaluated in hemodilution [23], surgery [24–29], and intensive care [11, 30]. Most studies have evaluated absolute accuracy while only a few studies have systematically evaluated trend accuracy, which appears to be more relevant in blood loss assessment. In our study, we verified that revision E sensors for SpHb monitoring had clinically acceptable absolute accuracy. Laboratory hemoglobin values vary depending on a variety of factors including sampling site. Typically laboratory hemoglobin is measured at the peripheral vein (macrocirculation) reflecting hemoglobin, while SpHb measures capillary (microcirculation) hemoglobin. Macrocirculation and microcirculation have different physiological responses to acute events, such as blood loss, which must be taken into consideration when making comparison to laboratory values [31, 32].

When SpHb values were compared to a reference laboratory device in our study, we found a higher standard deviation than what has been reported for Hb measurements from consecutive blood samples run on the same model lab device [33]. However, the standard deviation of SpHb to Hb in our study was similar to that found for Hb measurements from consecutive blood samples analyzed on two separate laboratory devices [34]. Regarding trend accuracy, the high correlation we observed indicates that increases and decreases in laboratory Hb were captured with the changes in SpHb. SpHb has the added benefit of not just calculating the differences or lack of differences between intermittent measurements, but also providing data continuously.

In addition to the primary and secondary aims of this study, our results demonstrate that the implementation of SpHb monitoring could also provide significant cost savings. A 2007 study from Shander et a1. [8] showed annual expenditures on blood and transfusion-related activities, for surgical patients only, ranged from 1.6 to 6 million dollars, or $522 to $1,183 per RBC unit. These estimates use activity based costs, which are the true cost of giving blood because they include not just the material cost of blood but also the direct costs to administer blood throughout the multistep procedure. Our results indicate a reduction of 0.9 units of blood per surgery or between $470 to $1,065 per patient monitored and $470,000 to $1,065,000 per 1,000 surgeries of the same type (Table 2). Note that this does not include the intra-hospital or even intra-country variability in cost of transfusion, nor the cost of devices and sensors.

Our study has some limitations worth noting. We used a convenience sample and did not randomize all patients after enrollment due to logistics of assigning them to the operating room with a sole SpHb monitor, but instead randomized which patient would be enrolled in the SpHb Group. This also led to an expected but unbalanced number of patients in both groups, as we were able to enroll multiple patients on the same day in the Control Group. Nonetheless, there was no bias in SpHb patient selection and there was no difference in baseline characteristics between groups. Therefore the differences in the SpHb Group can be attributed to the availability and use of SpHb monitoring. Furthermore, we were not able to blind the study given that clinicians had to make decisions in real-time data; this decreased the overhead delay in assessing hemoglobin concentrations, and consequently clinicians were able to start and stop transfusions in a timelier manner. While this may be viewed as a study bias, we view the difference of having a continuous data stream as the benefit of SpHb monitoring rather than a limitation of the study.

Finally, we were not able to collect post-surgery transfusion data or post-discharge information, which would facilitate longer-term assessment of our investigation. Also, our hospital’s blood transfusion practice may be different from other hospitals and that may limit transferability of our findings to other institutions. Unless clinical signs dictate otherwise, such as a massive bleeding event during surgery, it is standard practice in our hospital to initiate a hemoglobin measurement when EBL reaches approximately 15 % of estimated blood volume (EBV) and to initiate blood transfusion when the hemoglobin value is confirmed to be ≤l0 g/dL.

## Conclusion

Measurement of Hb is one of the most frequent and important assessments in patients undergoing major surgery and in patients admitted to the critical care unit. Unfortunately, invasive blood sampling and laboratory analysis only offer the perspective of a single point in time, while hemoglobin is in fact dynamic. This means that when the results of the “still picture” taken 5–50 min earlier become available, the “picture” may have changed. Our belief is that the real-time ability of SpHb [35] offers a “motion picture” that does not replace the metaphorical still picture, but supplements its inherently intermittent and delayed results. As shown in our study, the ability to observe the continuous trend in hemoglobin affects transfusion behavior, allowing earlier cessation of RBC transfusion as well as earlier consideration of initiation of RBC transfusion.

## List of abbreviations

RBC: Red blood cell
Hb: Laboratory hemoglobin
SpHb: Continuous and noninvasive hemoglobin monitoring with Pulse CO-Oximetry
ASA: American Society of Anesthesiologists
EBL: Estimated blood loss
EBV: Estimated blood volume
